# First‐Trimester Fetal Cardiac Function Measurements Using Spatio‐Temporal Image Correlation and Two Ultrasound‐Related Post‐Processing Methods: A Feasibility and Reproducibility Study

**DOI:** 10.1002/pd.6846

**Published:** 2025-07-09

**Authors:** K. Zandbergen, M. Rousian, Q. C. J. Griep, A. H. J. Koning, J. M. J. Cornette, B. R. Rebel, E. A. P. Steegers, A. G. M. G. J. Mulders

**Affiliations:** ^1^ Department of Obstetrics and Gynecology Erasmus MC University Medical Center Rotterdam the Netherlands; ^2^ Department of Pathology Erasmus MC University Medical Center Rotterdam the Netherlands; ^3^ Department of Pediatric Cardiology Erasmus MC Rotterdam the Netherlands

## Abstract

**Objective:**

To study the feasibility and reproducibility of two ultrasound (US) related post‐processing methods for first‐trimester fetal cardiac function assessment by ventricle volume measurements.

**Method:**

First‐trimester transvaginal Spatio‐Temporal Image Correlation (STIC) US datasets acquired between 11^+0^–13^+6^ weeks gestational age (GA) were used to perform fetal cardiac ventricle volume (FCVV) measurements in the end‐diastolic (EDVV) and end‐systolic (ESVV) phases using two methods: the manual segmentation method Virtual Organ Computed‐Aided AnaLysis (VOCAL) and (semi‐)automated volume measuring method Virtual Reality (VR). Reproducibility was assessed by calculating the intra‐, interobserver and intersystem agreement using intraclass correlation coefficients (ICCs) followed by Bland‐Altman plots.

**Results:**

25 STIC US datasets were selected. The mean GA was 13^+0^ weeks (SD 2.3 days) and mean crown‐rump length was 68.0 mm (range 61.0–75.6 mm). The intra‐ and inter‐observer agreement for both methods resulted in good to excellent agreement (ICCs > 0.85). Mean relative differences for all FCVV measurements were < 10.0%, except for the inter‐observer agreement of the VOCAL ESVV measurement (40.6%). The inter‐system agreement showed poor to moderate agreement (ICCs 0.32–0.75) and moderate to good agreement (ICCs 0.62–0.78) in terms of absolute agreement and consistency, respectively.

**Conclusion:**

FCVV measurements performed in STIC US datasets using VR are feasible and reproducible, specifically when compared to VOCAL.

## Summary

- What's already known about this topic?
  ◦ Antenatal identification of cardiovascular (dys)function may offer insight into cardiac adaptation and help to identify high‐risk populations. We assessed the feasibility and reproducibility of two ultrasound post‐processing methods (Virtual Organ Computer‐aided AnaLysis (VOCAL) and Virtual Reality (VR)) for first‐trimester fetal cardiac ventricle volume (FCVV) measurements.
- What does this study add?
  ◦ FCVV measurements using VR are feasible and reproducible and could serve as novel imaging markers for fetal cardiac function during early pregnancy.

## 1 Introduction

Cardiovascular diseases (CVD) are worldwide the leading cause of morbidity and mortality [1]. The developmental origins of health and disease (DOHaD) paradigm postulates that a suboptimal in‐utero environment can cause structural changes in key organ systems such as cardiac tissue [2, 3, 4]. Cardiac remodeling may affect long‐term cardiac function, resulting in an increased risk of CVD in later life [5]. The exact pathophysiology of in‐utero cardiac remodeling remains unknown. Developing reliable first‐trimester measurements reflecting fetal cardiac function holds potential for serving as early markers for (sub)clinical myocardial dysfunction. However, despite technological advancements, prenatal assessment of first‐trimester fetal cardiac function still has severe limitations [6].

Fetal cardiac ventricular volume (FCVV) is proposed as a promising marker for cardiac function; however, it is largely based on geometric assumptions made on two‐dimensional (2D) ultrasound (US) data [7]. As a volume consists of three dimensions, the reliability of these models has therefore been questioned [8]. Nevertheless, non‐gated acquisition of the heart may introduce artifacts due to movements (i.e., myocardial contractions) in the reconstructed volumetric data. Spatio‐Temporal Image Correlation (STIC) enables the reconstruction of a 3D cineloop sequence of the fetal cardiac cycle [9]. Subsequently, several attempts have been made to design a feasible method for FCVV measurements using STIC volume datasets [6, 8, 10]. A method for segmentation of FCVV measurements is Virtual Organ Computer‐aided AnaLysis (VOCAL). However, to date, this method appears to be not reproducible in the first trimester of pregnancy [6, 8, 10, 11]. Another notable limitation of VOCAL is the reliance on a flat screen for visualizing 3D US data, which still impedes the perception of depth in the third dimension. At the Erasmus Medical Center, the use of an in‐house developed volume rendering application called V‐Scope is used to display 3D US data as holograms. Virtual Reality (VR) facilitates unlimited magnification and rotation of the acquired 3D data along all three axes, as well as (semi‐)automated segmentation of various volumetric measurements of fetal structures in early pregnancy in a feasible and reproducible manner [12, 13, 14, 15, 16, 17]. VR techniques, initially developed for research purposes, have now also been integrated into the outpatient clinical setting [18].

Therefore, our aim is to study the feasibility and reproducibility of two US related post‐processing methods (VOCAL and VR) for first‐trimester FCVV measurements using STIC volume datasets.

## 2 Methods

### 2.1 Study Population

For this study, a subset of STIC volume datasets from first‐trimester high risk pregnancies between 11^+0^–13^+6^ weeks' GA were selected based on their quality. High risk was defined as, or in case of: (1) fetus with one first‐degree relative with structural abnormality not based on a diagnosed genome abnormality, (2) fetus with two second‐degree or other further relatives with a similar anomaly, (3) maternal disease (e.g., diabetes mellitus), (4) pregnancy conceived after IntraCytoplasmic Sperm Injection (ICSI), (5) substance use and/or use of teratogenic medication/drugs or/and (6) monozygotic multiple pregnancy. Hospital or midwifery charts were obtained to complete follow‐up on all participants. In our cohort, only datasets from fetuses without congenital anomalies were selected. All participants provided written informed consent as a component of the VR FETUS study. The extensive VR FETUS study design has previously been published [18].

### 2.2 Ethical Approval

The study protocol was approved by the Erasmus MC Institutional Review Board (MEC‐2004‐227) and all participating women and their partners signed written informed consent at enrollment (reference number: NL58563.078.16).

### 2.3 Acquisition of STIC Volume Datasets

The first‐trimester US examinations were performed on a Voluson E8 or E10 (GE Healthcare, Austria) machine using a 6–13 MHz high frequency transvaginal (TV) transducer and a 2–6 MHz transabdominal (TA) transducer. Examinations followed a standardized protocol (Figure S1) and international guidelines on safe use of US in the first trimester of pregnancy, minimizing total scanning time (ALARA‐principle) [19]. During ultrasound examination 3D‐ and STIC volume datasets from the fetus and fetal heart were obtained by experienced sonographers. At least three STIC volume datasets including color Doppler imaging (CDI) were acquired. First, an optimal fetal four‐chamber view was obtained. Acquisition time was set between 7.5 and 15 s to minimize motion and the angle of acquisition ranged between 15 and 20°. The images were stored as Cartesian datasets on a secure storage platform using specialized 3D software (4D View, version 5.0, GE Medical Systems).

Per pregnancy, the quality of the acquired and stored STIC volume datasets was assessed according to a scoring system based on the presence or absence of motion artifacts (yes/no), shadow artifacts (yes/no), volume completeness (complete/incomplete) and blurriness (yes/no). This generated a total score of 0–4. Score 0 was considered as poor quality, score 1 as low quality, score 2 as medium quality, score 3 as good quality and score 4 as high quality. Since we were conducting a feasibility study, only STIC volume datasets of good (3) and high (4) quality score were selected for assessment. Per pregnancy, the STIC US dataset with the highest score was used for further assessment. In cases of equal quality scores, the first acquired STIC US dataset was used. For each selected STIC US dataset, the following additional information of the pregnancy at the moment of ultrasound examination was collected: maternal age, height, weight, ethnicity, parity, folic acid supplement use (yes/no), smoking (yes/no), mode of conception, reason for prenatal diagnosis, fetal gestational age (GA), crown‐rump length (CRL), fetal heart rate (HR), and US approach.

### 2.4 Offline STIC Ultrasound Dataset Assessment

Fetal cardiac function is reflected by stroke volume (SV), which represents the amount of blood ejected per heartbeat, and can be calculated by subtracting the end‐diastolic ventricle volume (EDVV) from end‐systolic ventricle volume (ESVV) [20]. A STIC US dataset consists of a time series of volumes, which can be played like a video clip. Consequently, the technique allows the playback to be stopped at any time (frame) in the cardiac cycle. To accurately compare the measurements from the different methods, only measurements obtained from the same framerate in the acquired STIC volume datasets (representing the ESVV and EDVV) were employed for further analysis.

FCVV measurements were performed in both gray‐scale mode and in color Doppler mode. In order to pursue the most accurate method (largest precision of a cardiac ventricle volume), measurements were performed in gray‐scale mode (VOCAL and VR‐GS). To introduce a practical and acceptable method in clinical settings, ventricle volume measurements were also performed in color Doppler mode (VR‐CD). For FCVV measurements performed on STIC volume datasets in gray‐scale mode, the color Doppler imaging (CDI) signal was intentionally deactivated. Comparing FCVV measurements performed in VR‐GS mode with those in VR‐CD mode is feasible only when conducted at the same framerate within the cardiac cycle. Consequently, the end‐diastolic (ED) and end‐systolic (ES) phases (along with their corresponding framerates) were determined based on the minimum and maximum presence of the CDI signal (based on atrioventricular (AV) blood flow).

### 2.5 FCVV Measurements Performed With VOCAL

VOCAL allows measurements of the FCVV by manual tracing. For the assessment of the STIC volume datasets in VOCAL, measurements were performed using 4D‐View software version 17.1 (GE Medical Systems). 4D‐view enables visualization of the heart in three orthogonal planes at the same time and rotation of a plane around a fixed axis. Around this fixed axis, a sequence of six sections of the fetal cardiac ventricle is presented. FCVV measurements were obtained by manually tracing along the endocardial borders with a 30‐degree rotation angle. Based on these contours, a 3D reconstruction of the ventricle volume is made, and the volume is calculated automatically in cm^3^ [10]. To compare FCVV measurements from both methods (VOCAL and VR) FCVV measurements performed with VOCAL were converted into the same unit of measurement (mm^3^) of FCVV measurements performed using the VR method.

### 2.6 FCVV Measurements Performed With VR

The V‐Scope software allows (semi‐)automated measurements of FCVV using VR techniques. For the current study, FCVV measurements were performed using the I‐Wall. The I‐Wall is a single surface projection‐based immersive VR system installed at the Erasmus MC. This concept is essentially a single walled CAVE system and is also known as a PowerWall [21]. The V‐Scope software is being developed at the Erasmus MC and is a volume rendering application used to create an interactive “hologram” of the US dataset [12]. This hologram can be manipulated by using a virtual pointer that is controlled by a wireless joystick, facilitating the assessment of STIC volume datasets from multiple angles and in any eligible plane [22]. Further demonstration of the clinical application of 3D VR is provided in Video S1. For offline VR assessment, V‐Scope implements a semi‐automatic segmentation algorithm in which the user places a seed point in the targeted area. The algorithm then selects the surrounding object or tissue controlled by a number of user selectable thresholds.

**FIGURE 1:**
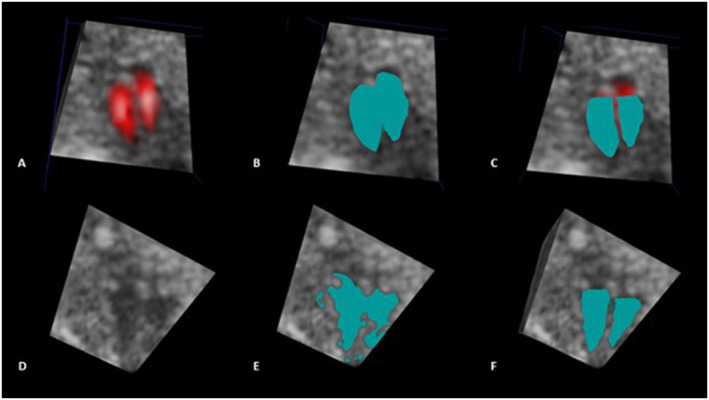
Infographic (panel A to F) showing how Virtual Reality (VR) was applied to perform fetal cardiac ventricle volume (FCVV) measurements. Panel (A–C) FCVV measurements performed in color Doppler mode (VR‐CD). (A) A Spatio‐Temporal Image Correlation (STIC) ultrasound (US) dataset displayed in color Doppler (CD) mode (based on atrioventricular blood flow). (B) FCVV measurement performed using VR in color Doppler (VR‐CD) mode. The colored (cyan) regions indicate the segmented region of the cardiac ventricle after using an automated segmentation algorithm based on a pre‐set number of voxel (red or blue‐) value thresholds. (C) The colored (cyan) regions indicate the segmented region of the cardiac ventricle after manually removing incorrect (un)segmented voxels from the segmented region in “B”. Panel (D–F) FCVV measurements performed in gray‐scale mode (VR‐GS). (D) A STIC US dataset displayed in gray‐scale (GS) mode. (E) FCVV measurements performed using VR in gray‐scale (VR‐GS) mode. The colored (cyan) regions indicate the segmented region of the cardiac ventricle after using an automated segmentation algorithm based on a pre‐set number of voxel (gray‐)value thresholds. (F) The colored (cyan) regions indicate the segmented region of the cardiac ventricle after manually adding or removing incorrect (un)segmented voxels from the segmented region in “E”.

If needed, multiple “seed points” can be used to grow the segmented region until the entire targeted area is segmented. A spherical free hand “paint brush” with a pre‐defined fixed diameter can then be used to correct the semi‐automatic segmentation by manually adding or removing voxels from the segmented region. In this way, incorrectly unsegmented areas of the cardiac ventricle were added, while incorrectly segmented regions (such as the surface of the atria, outflow tracts, and interventricular septum) were removed. In our study, the targeted areas comprised the hypoechoic region of the fetal cardiac ventricle in FCVV measurements performed in VR‐GS mode and the blue and/or red for measurements performed in VR‐CD mode (Figure 1). Based on the obtained segmentation, the ventricular volume was calculated automatically in mm^3^. Video 1 demonstrates the performance of an FCVV measurement using the VR method of an STIC US dataset in VR‐CD mode. Video 2 demonstrates performing an FCVV measurement using the VR method of an STIC US dataset in VR‐GS mode.

**VIDEO 1:** FCVV measurement performed with VR‐CD method. To view this video in the full‐text HTML version of the article, please visit https://onlinelibrary.wiley.com/doi/10.1002/pd.6846.

**VIDEO 2:** FCVV measurement performed with VR‐GS method. To view this video in the full‐text HTML version of the article, please visit https://onlinelibrary.wiley.com/doi/10.1002/pd.6846.

All measurements were performed by two independent researchers (Q.G. and K.Z.) who were blinded to each other's results. Observer 1 (Q.G.) performed all series of measurements at two different time points with an interval of 2 weeks' blinded from the initial measurements to prevent recall bias. To minimize potential measurement errors, all measurements were performed in triplicate, of which the mean was used for further analysis. Finally, the duration of time required to obtain FCVV measurements in both methods (VOCAL and VR) was monitored.

### 2.7 Statistical Analysis

**FIGURE 2:**
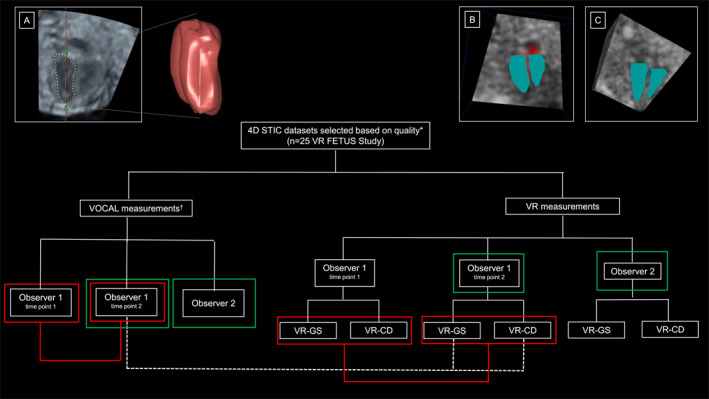
The Flowchart illustrates the acquisition of the fetal cardiac ventricle volume (FCVV) measurements using the Virtual Organ Computer‐aided AnaLysis (VOCAL) method and the Virtual Reality (VR) method. Panel (A) depicts an FCVV measurement using VOCAL, after manual tracing of the endocardial borders in gray‐scale mode and a three‐dimensional (3D) rendering of the heart. Panel (B) depicts an FCVV measurement using VR performed in color Doppler mode (VR‐CD). Panel (C) depicts an FCVV measurement using VR performed in gray‐scale mode (VR‐GS). The red contouring indicates the intra‐observer variability for observer 1 at time points 1 and 2. The green contouring indicates inter‐observer variability between observer 1 and observer 2. The white dotted lines indicate the inter‐system variability. *quality was based on the presence of: motion artifacts (yes/no), shadow artifacts (yes/no), volume completeness (complete/incomplete) and blurriness (yes/no). Only STIC US datasets with good and high quality scores were selected for assessment. † all VOCAL measurements are performed in gray scale datasets. CD, color doppler; GS, grayscale; STIC, spatio‐temporal image correlation; VOCAL, virtual organ computer‐aided analysis; VR, Virtual Reality.

Statistical analysis carefully followed the steps described previously by Bland and Altman [23]. First, individual FCVV measurements were plotted with the line of equality and corresponding Pearson's correlation coefficients (R‐values) to obtain an impression of the degree of agreement. Second, the consensus between (intra‐observer agreement) and among observers (inter‐observer agreement) and the systems used (inter‐system agreement) was analyzed by calculating intraclass correlation coefficients (ICCs). Additionally, median FCVV measurements including range, mean differences, mean relative differences with standard deviation and 95% limits of agreement for all FCVV measurements were calculated to quantify variability [23, 24]. For the intra‐observer agreement, the first series of measurements were compared with the second series of measurements of observer 1. For the inter‐observer agreement, the second series of measurements of observer 1 was compared with the measurements of observer 2. For the inter‐system agreement, the measurements of observer 1 performed using the post‐processing method VOCAL were compared with the FCVV measurements of observer 1 performed using the VR (in different modes: VR‐CD & VR‐GS) method. Figure 2 represents a flowchart of FCVV measurements using the VOCAL and VR performed by both observers and illustrates the intra‐observer, inter‐observer and inter‐system agreement. ICC values > 0.90 indicate excellent agreement, values between 0.75–0.9 indicate good agreement, values between 0.5 and 0.75 indicate moderate agreement and values < 0.5 are indicative for poor agreement. To visualize the intra‐observer, inter‐observer and inter‐system agreement, Bland‐Altman plots were composed by calculating the mean difference and 95% limits of agreement (mean difference [%] ± 1.96SD).

All analyses were performed using SPSS software (version 28.0.1.0; SPSS Inc., Chicago, IL, USA) and *p*‐values < 0.05 were considered statistically significant.

## 3 Results

For this study, a subset of STIC volume datasets from 25 out of 186 first‐trimester pregnancies between 11^+0^–13^+6^ weeks' GA were selected based on their quality. Table 1 provides an overview of the maternal and fetal background characteristics of the 25 selected pregnancies at the time of US dataset acquisition. For the 25 selected pregnancies, the quality of the STIC volume datasets was high (score 4) in 14 (56%) and good (score 3) in 11 (44%) cases. Mean GA at ultrasound examination was 13^+0^ weeks (range 12^+5^–13^+6^ days) and mean CRL was 69.1 mm (range 61.0–75.6 mm). In 23 (92%) women the datasets were recorded using transvaginal (TV) US and in 2 (8%) women a transabdominal (TA) US approach was used.

**TABLE 1:** Pregnancy characteristics of the selected STIC US datasets (*n* = 25).

Characteristics	Mean (SD)/*n* (%)	[95% CI for mean]
Maternal		
Maternal age (years)	28.5 (4.3)	[26.7; 30.3]
Body mass index (kg/m^2^)	24.3 (4.1)	[22.5; 26]
Geographic origin		
West‐European	21 (84%)	
Other	4 (16%)	
Nulliparous	15 (60%)	
Folic acid supplement use, yes	14 (56%)	
Smoking, yes (cigarettes)	8 (32%)	
Mode of conception		
Natural	21 (84%)	
IVF/ICSI/OI	4 (16%)	
Reason of prenatal diagnostics		
1st degree family member congenital anomaly	18 (72%)	
Teratogenic drug use	2 (8%)	
Maternal disease	4 (16%)	
Mode of conception: IVF/ICSI	1 (4%)	
Fetal		
Gestational age (weeks ^+ days^)	13^+1^ (2.3 days)	[13^+0^;13^+2^]
Crown rump length (CRL (mm))	69.0 (4.5)	[67.1; 70.9]
Fetal heart rate (bpm)^a^	157 (7.2)	[154; 160]
Ultrasound approach		
Transabdominal	2 (8%)	
Transvaginal	23 (92%)	
Quality score STIC US datasets		
Good (score 3)	11 (44%)	
High (score 4)	14 (56%)	

Abbreviations: Bpm, beats per minute; CI, confidence interval; ICSI, intracytoplasmic sperm injection; IVF, in vitro fertilization; OI, ovulation induction; STIC, spatio‐temporal image correlation; US, ultrasound.

^a^ Fetal heart rate acquired during STIC US dataset acquisition.

All FCVV measurements were plotted with a line of equality with corresponding Pearson's correlation coefficients (*R*‐values) as shown in Figures S2–S4. FCVV measurements performed using both post‐processing methods; VOCAL and VR (in different modes: VR‐CD and VR‐GS) showed strong correlations (*R* = 0.79–0.99) for the intra‐observer and inter‐observer agreement. The inter‐system agreement showed correlation coefficients ranging from *R* = 0.48 to *R* = 0.75 (which corresponds to moderate to strong correlation).

Table 2 provides an overview of all median FCVV measurements and (relative) mean differences with corresponding intra‐observer and inter‐observer variability parameters (ICC and 95% limits of agreement). The intra‐observer and inter‐observer variability parameters for the FCVV measurements using VOCAL showed good to excellent agreement with ICC values > 0.85 (range 0.85–0.96). The intra‐observer and inter‐observer variability parameters for the FCVV measurements using VR showed excellent agreement with ICC values > 0.93 (range 0.93–0.99). Mean relative differences for all FCVV measurements were < 10.0%, with the exception of the inter‐observer agreement of the VOCAL ESVV measurement (40.6%).

**TABLE 2:** Intra‐observer and inter‐observer variability of first‐trimester fetal cardiac ventricle volume measurements using VOCAL and VR (VR‐CD and VR‐GS) (*n* = 25).

Used method/mode	Median		Intra‐observer agreement					Inter‐observer agreement				
			ICC	Mean difference		Relative mean difference		ICC	Mean difference		Relative mean difference	
	(mm^3^)	[Range]		(mm^3^)	[95% LoA]	(%)	[95% LoA]		(mm^3^)	[95% LoA]	(%)	[95% LoA]
VOCAL												
EDVV	25.7	[10.3; 85.7]	0.96	−2.6	[−11.9; 6.8]	−5.9	[−36.9; 25.0]	0.92	−0.1	[−14.0; 13.9]	3.6	[−48.1; 55.3]
ESVV	12.7	[4.3; 48.0]	0.93	−0.2	[−7.7; 7.4]	−2.1	[−46.5; 42.3]	0.85	0.9	[−7.5; 14.4]	40.6	[−105.3; 186.4]
VR												
(VR‐CD)												
EDVV	51.7	[28.8; 130.0]	0.98	−1.0	[−13.4; 11.5]	0.2	[−22.7; 23.0]	0.98	−1.1	[−13.3; 11.0]	−0.5	[−21.8; 20.8]
ESVV	18.9	[3.3; 63.3]	0.99	−1.5	[−5.6; 2.6]	−6.1	[−22.5; 10.4]	0.98	1.5	[−5.5; 8.5]	7.6	[−30.9; 46.1]
(VR‐GS)												
EDVV	51.7	[14.9; 91.1]	0.96	−1.3	[−15.8; 13.1]	−1.7	[−26.9; 23.5]	0.93	−3.8	[−20.4; 12.8]	−7.0	[−36.6; 22.6]
ESVV	27.5	[8.5; 60.6]	0.98	−0.7	[−8.0; 6.6]	−1.7	[−30.8; 27.4]	0.93	0.6	[−12.9; 14.1]	2.6	[−43.2; 48.4]

Abbreviations: EDVV, end‐diastolic ventricle volume; ESVV, end‐systolic ventricle volume; ICC, intraclass correlation coefficient; LoA, limits of agreement; VOCAL, virtual organ computer‐aided analysis; VR, Virtual Reality; VR‐CD, VR in color Doppler; VR‐GS, VR in gray‐scale.

FCVV measurements with the smallest limits of agreement (LoA) were performed in VR‐CD mode for both intra‐ and inter‐observer agreement. Intra‐observer LoA (VR‐CD ESVV) ranged from −22.5% to 10.4% difference from the mean, while inter‐observer LoA (VR‐CD EDVV) ranged from −21.8% to 20.8% of the mean. ESVV measurements performed with VOCAL had the widest LoA for both intra‐ and inter‐observer agreement, ranging from; −46.5%–42.3% and from −105.3% to 186.4%, respectively.

Table 3 shows the inter‐system agreement for the FCVV measurements performed using the post‐processing methods VOCAL and VR (in different modes: VR‐CD and VR‐GS).

**TABLE 3:** Inter‐system variability of first‐trimester fetal cardiac ventricle volume measurements using VOCAL and VR (VR‐CD and VR‐GS) (*n* = 25).

Used method/mode	ICC abs	ICC con	Inter‐system agreement			
			Mean difference		Relative mean difference	
			(mm^3^)	[95% LoA]	(%)	[95% LoA]
VOCAL versus VR‐CD						
EDVV	0.42	0.78	−30.3	[−60.3; −0.3]	−51.7	[−83.5; −19.9]
ESVV	0.62	0.71	−7.0	[−25.9; 11.9]	−9.4	[−174.8; 156.0]
VOCAL versus VR‐GS						
EDVV	0.32	0.67	−28.3	[−57.9; 1.3]	−50.0	[−84.6; −15.4]
ESVV	0.33	0.62	−16.5	[−37.9; −4.9]	−53.5	[−97.3; −9.7]
VR‐CD versus VR‐GS						
EDVV	0.75	0.74	−2.0	[−37.5; 33.4]	2.9	[−70.5; 76.4]
ESVV	0.62	0.74	9.5	[−11.8; 30.7]	95.6	[−155.8; 347.0]

Abbreviations: EDVV, end‐diastolic ventricle volume; ESVV, end‐systolic ventricle volume; ICC, intraclass correlation coefficient; ICC Abs, intraclass correlation coefficients in terms of *absolute agreement*; ICC Con, intra‐class correlation coefficients in terms of *consistency*; LoA, limits of agreement; VOCAL, virtual organ computer‐aided analysis; VR, Virtual Reality; VR‐CD, VR in color Doppler; VR‐GS, VR gray‐scale.

The intersystem agreement resulted in a poor to moderate agreement (ICC values ranging from 0.32 to 0.75) in terms of absolute agreement and in a moderate to good agreement (ICC values ranging from 0.62 to 0.78) in terms of consistency. Mean relative differences varied from 3% to 96%.

The LoA of the inter‐system agreement ranged from the smallest limit from −84.6% to 15.4% difference from the mean (VR‐GS vs. VOCAL EDVV) to the widest limit from −155.8 to 347.0 difference from the mean (VR‐CD vs. VR‐GS ESVV).

Figures S5‐S7 shows Bland and Altman plots for the mean relative differences in FCVV measurements (%) among or between observers and between the different used methods in proportions against the mean. The limits of agreement (±2 SD) are plotted in the figures.

The average time required to perform a FCVV measurement was 1.5 min, 2.5 and 4.5 min using the post‐processing methods VOCAL, VR‐CD and VR‐GS respectively.

## 4 Discussion

We demonstrated that first‐trimester FCVV measurements performed in STIC volume datasets using the post‐processing method VR appeared to be more reproducible compared with VOCAL. When comparing FCVV measurements performed with the VR method in VR‐CD mode versus VR‐GS mode, VR‐CD demonstrates greater reproducibility and therefore appears to be the most usable method. The distributions in the Bland‐Altman plots suggest that differences in measurements of smaller fetal cardiac ventricle volumes result in a proportionally wider range for the limits of agreement, thus decreasing overall reproducibility. VOCAL was the least time‐consuming method for FCVV measurements, followed by VR‐CD mode and then VR‐GS mode.

Our findings are likely attributable to the use of the optimal display modality, Virtual Reality (VR), for 4D visualization and interaction with STIC US data. The enhanced utility of measurements performed in VR‐CD mode compared to VR‐GS mode may be attributed to the larger automated component in VR‐CD, which is based on color Doppler signals derived from atrioventricular (AV) blood flow. Consequently, the reproducibility of the VR‐GS mode is limited due to its reliance on semi‐automated segmentation. Utilization of CDI signal for cardiac ventricular volume measurements depends on the presence of AV blood flow. Strictly speaking, blood flow over the AV valves ceases during both end‐diastolic and end‐systolic phases. As a result, the frames used for analysis in our study do not correspond with the frames that most accurately represent the end‐diastolic (ED) and end‐systolic (ES) phases in the cardiac cycle but may occur a fraction earlier. However, given our focus on feasibility and reproducibility rather than accuracy, this discrepancy is unlikely to impact our findings.

When comparing both methods, the absolute values of the FCVV measurements obtained using VOCAL appeared to be consistently smaller compared to the absolute values obtained using VR. Currently, there is no established in‐utero gold standard for obtaining these cardiac volumes. Therefore, we cannot determine the most accurate method. An explanation for the absolute differences in measurements could be that a ventricle volume measurement using VOCAL is derived by manually tracing the endocardial border around a “fixed” axis in a multiplanar mode. This means that the program interpolates the endocardial border between the rotational steps, which can lead to an underestimation of the enclosed volume. The application of VR enables unlimited rotation of the acquired STIC volume datasets around all three axes, allowing for a more accurate identification of the ventricular borders up to single voxel resolution. Furthermore, VOCAL calculates the volume in cubic centimeters (cm^3^), whereas VR employs cubic millimeters (mm^3^). Due to the minute size of the assessed structures, the utilization of cm^3^ results in reduced precision, which could be seen as a limitation. Hence, it is important to acknowledge that both methods do not agree closely and cannot be used interchangeably.

The main strength of our study is that, in contrast to the previously used 2D measurements, both VR and VOCAL avoid the use of geometric assumptions for estimating the ventricular volume. In addition, we consider the possibility of post‐processing of the acquired US datasets as an important value of our study. Offline assessment enables the investigator to evaluate the acquired and stored US datasets without any restriction of time and minimizes the exposure time of US examination.

During dataset acquisition, a strict US protocol was followed, experienced sonographers were involved and transvaginal scanning was preferred over transabdominal scanning. In the pursuit of developing a reproducible measurement, the use of a well‐designed and standardized protocol for performing measurements is obligatory and can therefore be seen as a strength in this context.

The process of quality selection may be perceived as a limitation as it has the potential to introduce selection bias. However, due to the selection of only high‐quality STIC volume datasets, we were able also to report on feasibility. In addition, it must be taken into account that the STIC volumes were obtained during a complete fetal ultrasound evaluation without the performance of targeted ultrasound examination of the heart alone. We are convinced that targeted scanning of the fetal heart only will further increase the quality of the STIC dataset and therefore the reproducibility. As our study population was recruited from a tertiary referral center, the results cannot be fully extrapolated to serve as a reference for the general population. Another potential drawback is the limited clinical applicability of both methods. Despite their commercial availability and potential value in healthcare, the lack of integration of VR and VOCAL techniques into available US devices hinders the implementation of cardiac function measurements in clinical practice. One possible reason for this is that, although the system appears user‐friendly, there is a learning curve required to perform measurements using the I‐Wall. Additionally, it may be considered a system that not all institutions have the financial resources to acquire or maintain. To facilitate the implementation of VR in an outpatient clinical setting, a VR desktop system ‐ using the same V‐Scope software as used for the I‐Wall ‐ was developed and validated [25]. The VR desktop system provides the same tools and functions as described for the I‐Wall but is inexpensive and has fewer logistical constraints, using a regular personal computer, a 3D monitor and an optical tracking system. As an alternative, VR‐based visualization can also be deployed remotely for expert consultations, providing access to specialized expertise without requiring the healthcare facility to directly acquire the system.

Repetitive measurements in population‐based cohort studies are needed to further validate our FCVV measurements and to assess their eligibility in determining potential (patho)physiological factors for fetal cardiac (dys)function.

Developing functional ultrasound markers, we also see an important role for the design of novel deep learning methods for the evaluation of US images, aiming to build an “artificially intelligent” screening instrument for the detection of cardiovascular anomalies [24]. In addition, future implementation of innovative measurements potentially allows us to identify (subclinical) changes in cardiac function during pregnancy.

Antenatal identification of cardiovascular dysfunction, which is associated with adverse conditions such as fetal growth restriction and fetal distress, may show promise in selecting high‐risk populations, providing opportunities for individualized management from early pregnancy onwards. Additionally, early monitoring of cardiac (dys)function could offer insights into the cardiac adaptations that occur during the transition from an intrauterine to an extrauterine environment. This ultimately enables the development of timely screening and preventive measures for cardiovascular risk factors and disease [4].

## 5 Conclusion

To our knowledge, this is the first study to demonstrate feasibility and reproducibility of first‐trimester FCVV measurements using STIC US data. FCVV measurements performed using VR as a post‐processing technique appeared to be more feasible and reproducible compared to VOCAL. Consequently, our results emphasize that FCVV measurements using VR could serve as a novel imaging marker for fetal cardiac function and ultimately provide insight into cardiac development during early pregnancy.

## Conflicts of Interest

The authors declare no conflicts of interest.

## Supporting information

Figure S1

Figures S2–S4

Figures S5–S7

Video S1

Figures S5–S7

## Data Availability

The data that support the findings of this study are available from the corresponding author upon reasonable request.
