# Supplementary material for: First‐Trimester Fetal Cardiac Function Measurements Using Spatio‐Temporal Image Correlation and Two Ultrasound‐Related Post‐Processing Methods: A Feasibility and Reproducibility Study
Source: Prenat Diagn. 2025 Jul 9;45(9):1130–8. doi: 10.1002/pd.6846 (PMC12322252; doi:10.1002/pd.6846)
Supplement: Supplementary file 1 — Figure S1 [file PD-45-1130-s001.docx]

**Supplemental figure S2**. Intra-observer agreement (measurements observer 1 at time point 1 vs. measurements observer 1 at time point 2) of FCVV measurements, plotted with line of equality (panel A-F).

**A) VR-CD EDVV B) VR-CD ESVV**


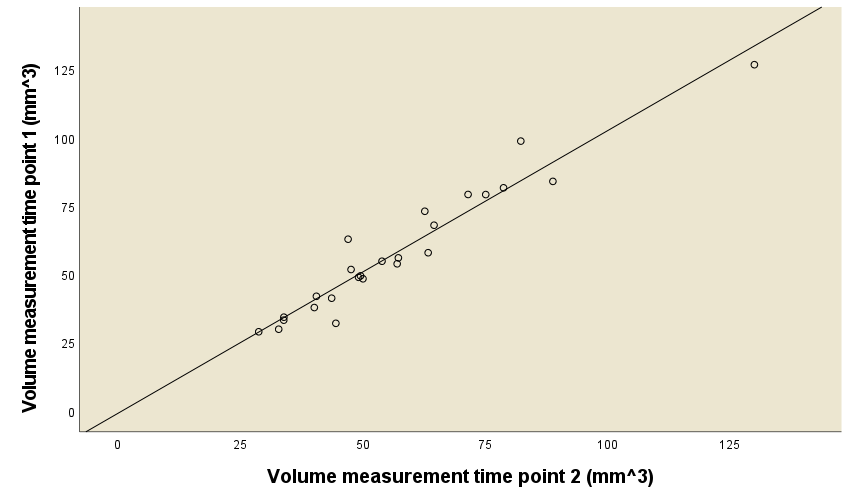

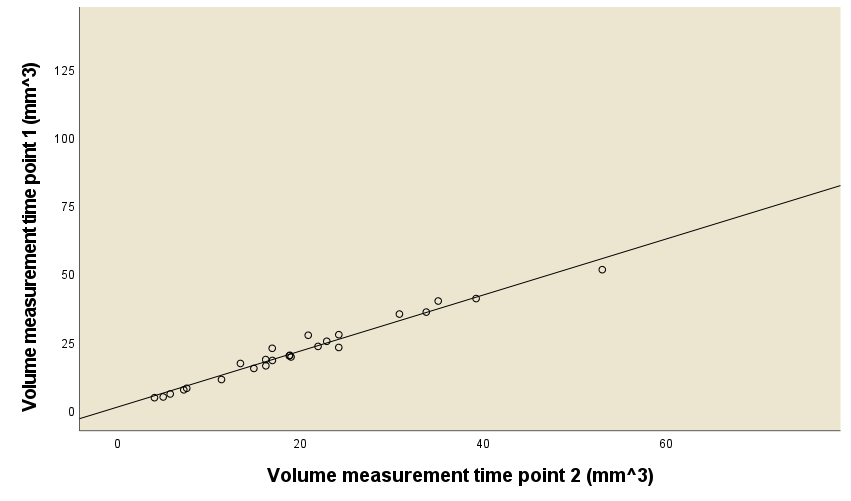


**Volume measurements time point 1 (mm^3^)**

**Volume measurements time point 2 (mm^3^)**

**Volume measurements time point 1 (mm^3^)**

**Volume measurements time point 2 (mm^3^)**

R = 0.99

R = 0.96

**C) VR-GS EDVV D) VR-GS ESVV**


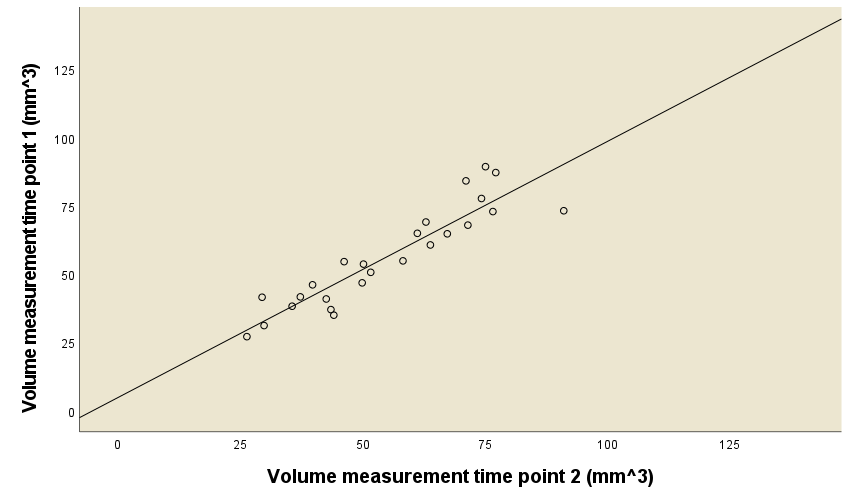

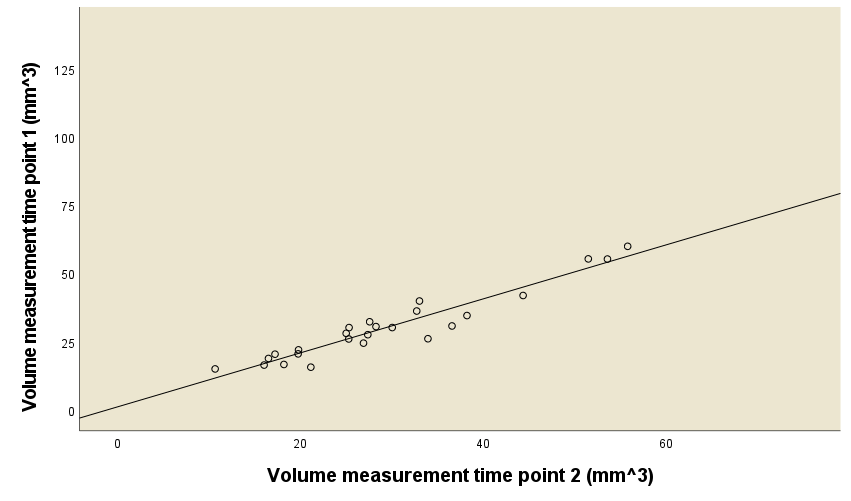


**Volume measurements time point 2 (mm^3^)**

**Volume measurements time point 1 (mm^3^)**

**Volume measurements time point 2 (mm^3^)**

**Volume measurements time point 1 (mm^3^)**

R = 0.95

R = 0.92

**E) VOCAL EDVV F) VOCAL ESVV**


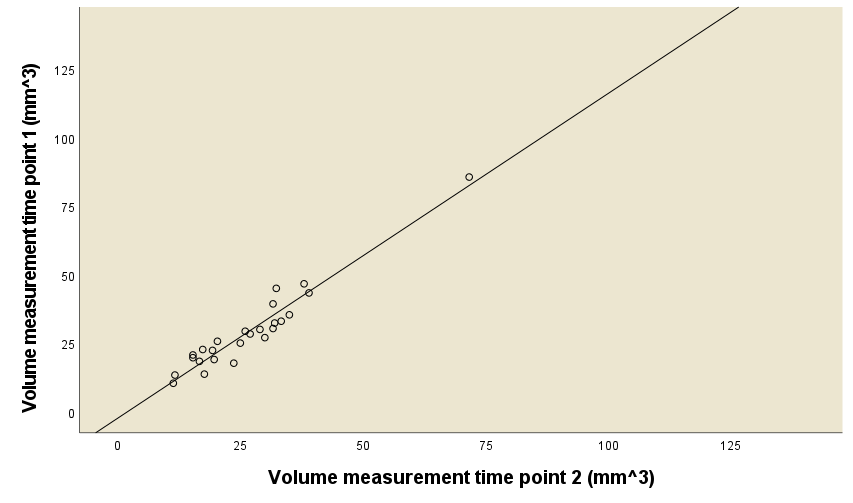

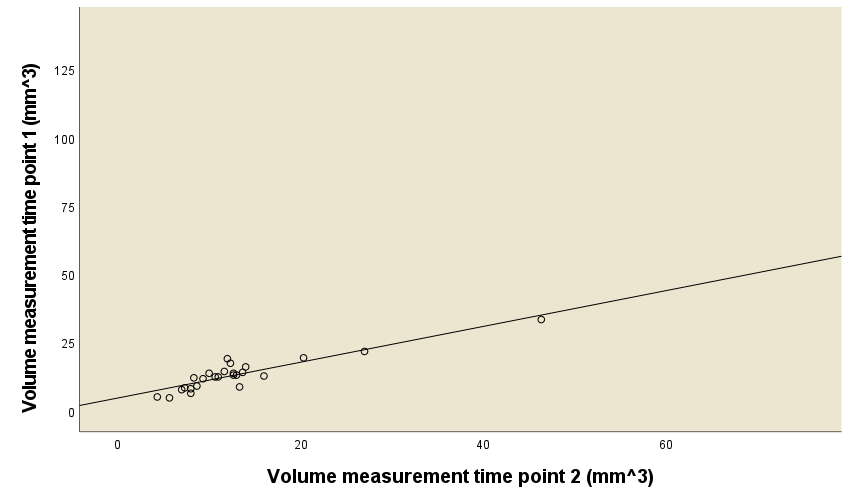


**Volume measurements time point 1 (mm^3^)**

**Volume measurements time point 2 (mm^3^)**

**Volume measurements time point 1 (mm^3^)**

**Volume measurements time point 2 (mm^3^)**

R = 0.91

R = 0.96

FCVV = fetal cardiac ventricle volume, VR = Virtual reality; VR-CD = VR color Doppler, VR-GS = VR gray-scale, EDVV = end-diastolic ventricle volume; ESVV = end-systolic ventricle volume; VOCAL = Virtual Organ Computer-aided AnaLysis, R = Pearson’s correlation coefficient**Supplemental figure S3**. Inter-observer agreement (measurements observer 1 at time point 2 vs. measurements observer 2) of FCVV measurements, plotted with line of equality (panel A-F).

**A) VR-CD EDVV B) VR-CD ESVV**


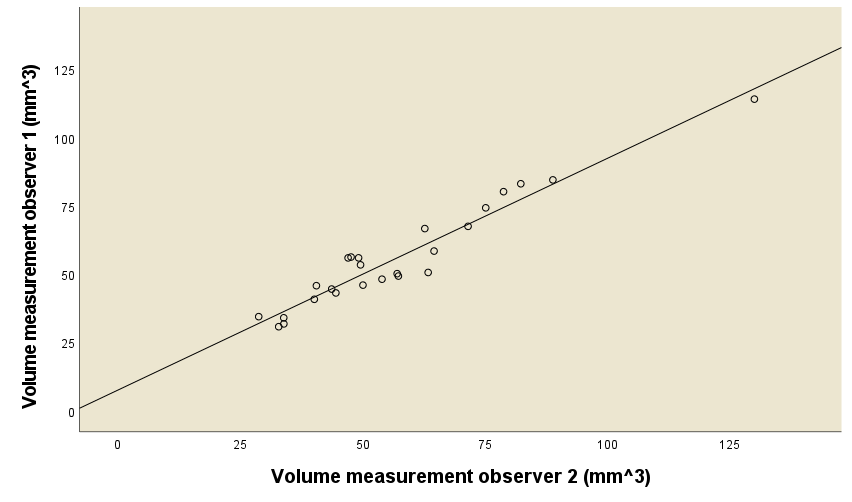

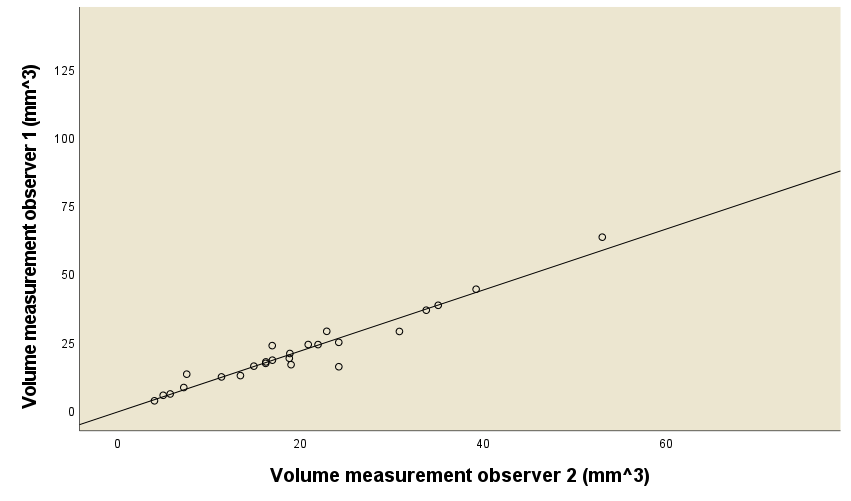


**Volume measurements observer 1 (mm^3^)**

**Volume measurements observer 2 (mm^3^)**

**Volume measurements observer 2 (mm^3^)**

**Volume measurements observer 1 (mm^3^)**

R = 0.97

R = 0.96

**C) VR-GS EDVV D) VR-GS ESVV**


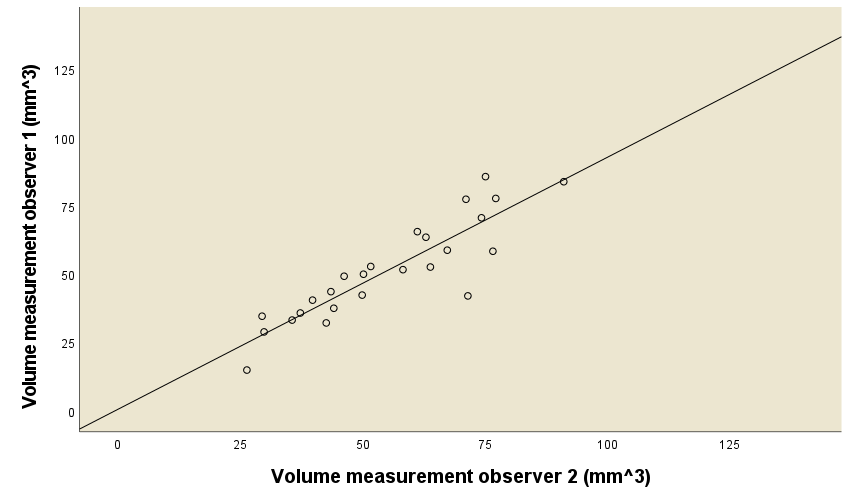

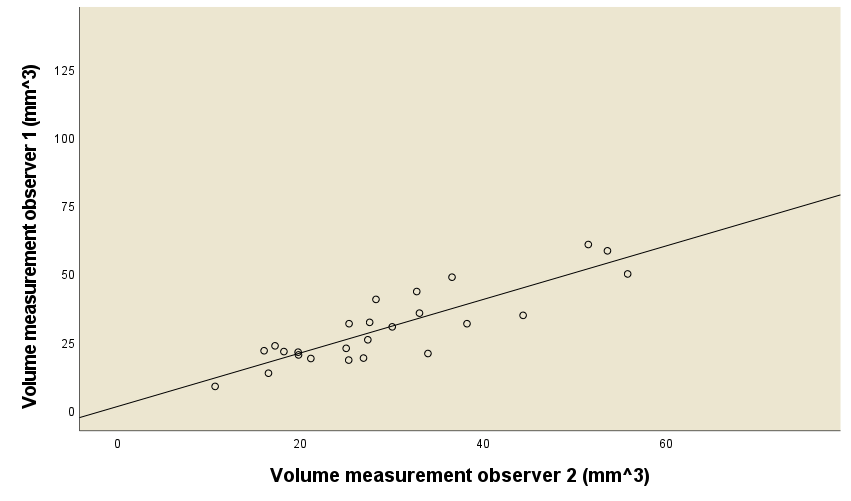


**Volume measurements observer 1 (mm^3^)**

**Volume measurements observer 2 (mm^3^)**

**Volume measurements observer 2 (mm^3^)**

**Volume measurements observer 1 (mm^3^)**

R = 0.86

**Volume measurement time point 2 (mm^3^)**

**Volume measurement time point 2 (mm^3^)**

R = 0.89

**E) VOCAL EDVV F) VOCAL ESVV**


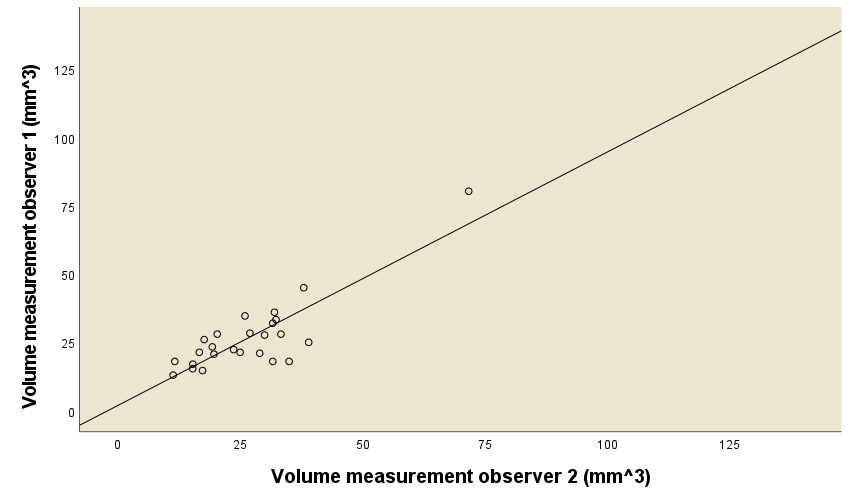

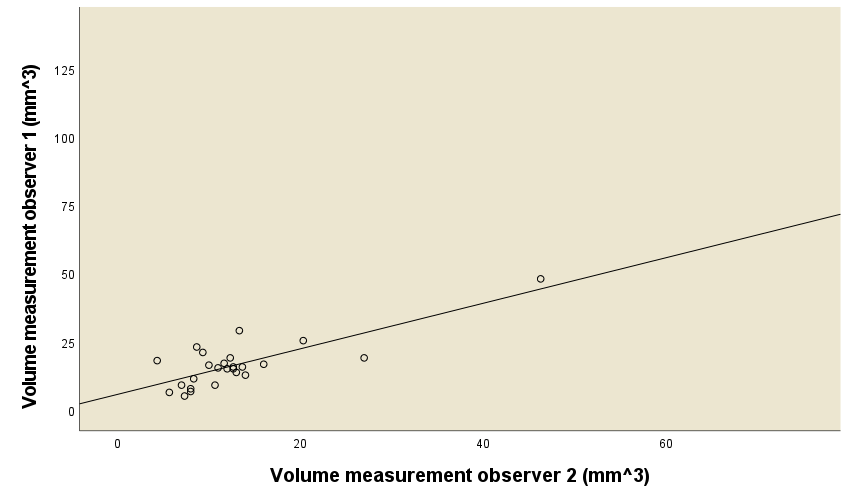


**Volume measurements observer 2 (mm^3^)**

**Volume measurements observer 1 (mm^3^)**

**Volume measurements observer 1 (mm^3^)**

**Volume measurements observer 2 (mm^3^)**

R = 0.79

R = 0.85

FCVV = fetal cardiac ventricle volume, VR = Virtual reality; VR-CD = VR color Doppler, VR-GS = VR gray-scale, EDVV = end-diastolic ventricle volume; ESVV = end-systolic ventricle volume; VOCAL = Virtual Organ Computer-aided AnaLysis, R = Pearson’s correlation coefficient

**Supplemental figure S4**. Inter-system agreement (measurements observer 1 at time point 2 vs. measurements observer 1 at time point 2) of FCVV measurements of plotted with line of equality and corresponding Pearson coefficient (panel A-F).

**A) VR-CD EDVV vs. VOCAL EDVV B) VR-CD ESVV vs. VOCAL ESVV**

**
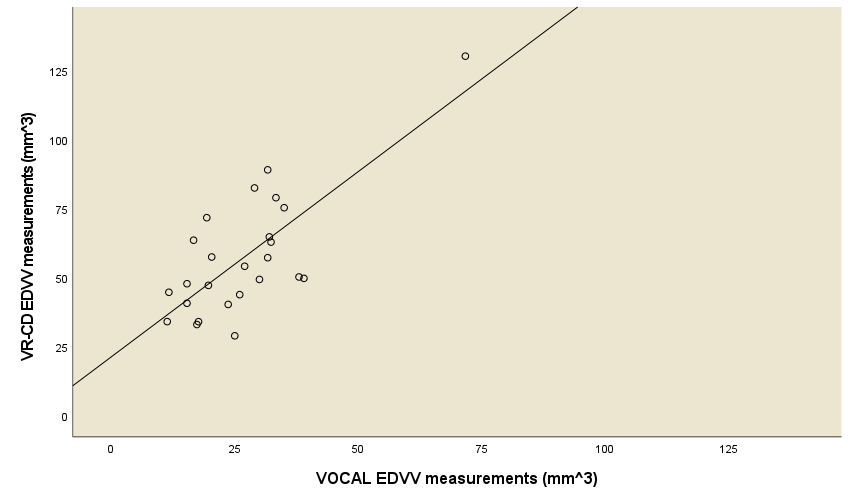

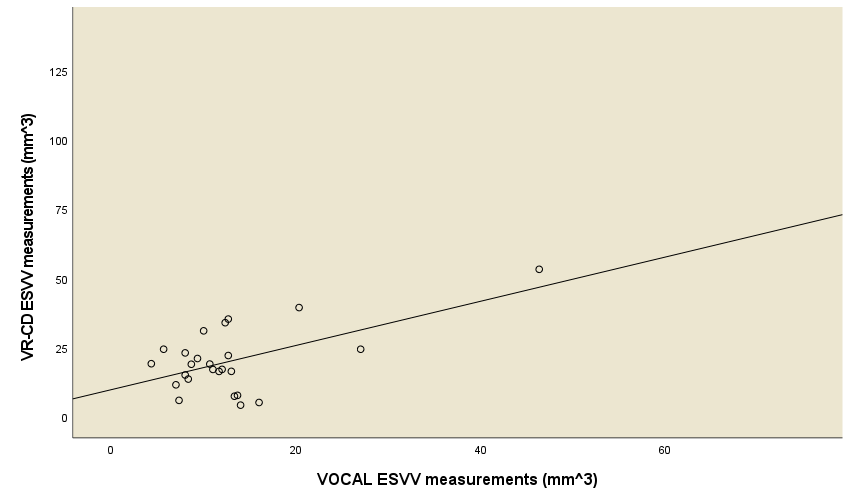
**

**VR-CD ESVV measurements (mm^3^)**

**VR-CD EDVV measurements (mm^3^)**

**VOCAL EDVV measurements (mm^3^)**

**VOCAL ESVV measurements (mm^3^)**

R = 0.58

R = 0.75

**C) VR-GS EDVV vs. VOCAL EDVV D) VR-GS ESVV vs. VOCAL ESVV**

**
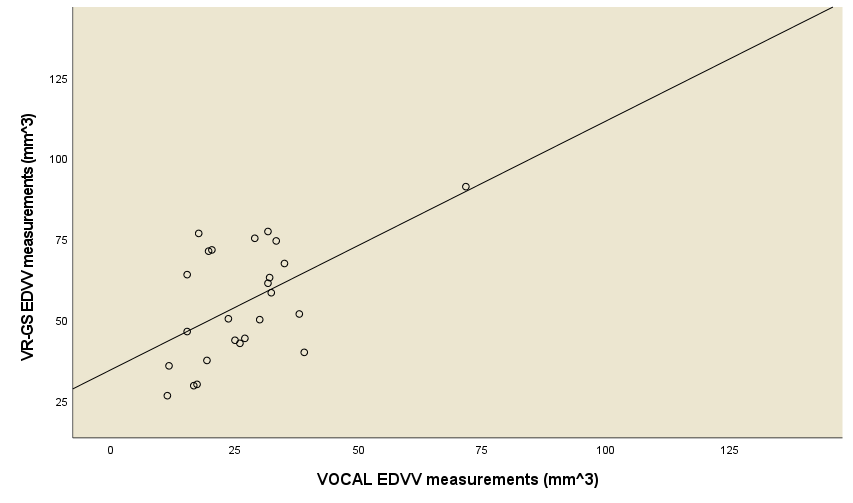

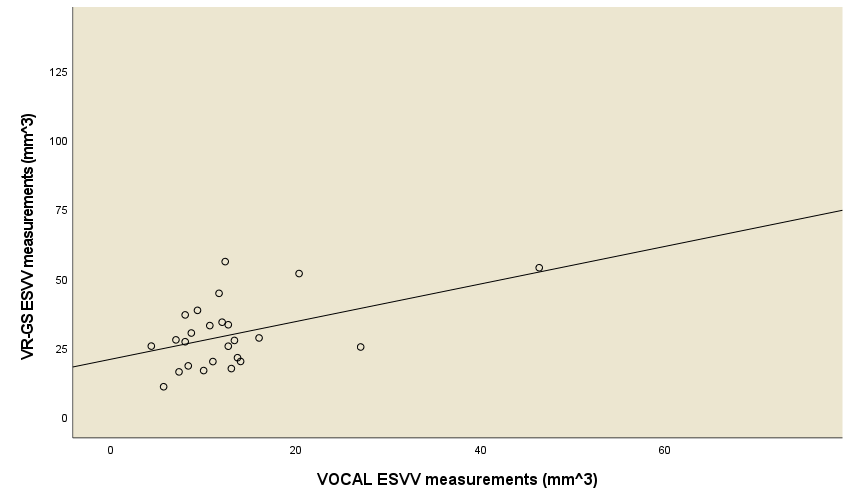
**

R = 0.48

**VOCAL ESVV measurements (mm^3^)**

**VR-GS ESVV measurements (mm^3^)**

**VR-GS EDVV measurements (mm^3^)**

**VOCAL EDVV measurements (mm^3^)**

R = 0.54

**E) VR-CD EDVV vs. VR-GS EDVV F) VR-CD ESVV vs. VR-GS ESVV**


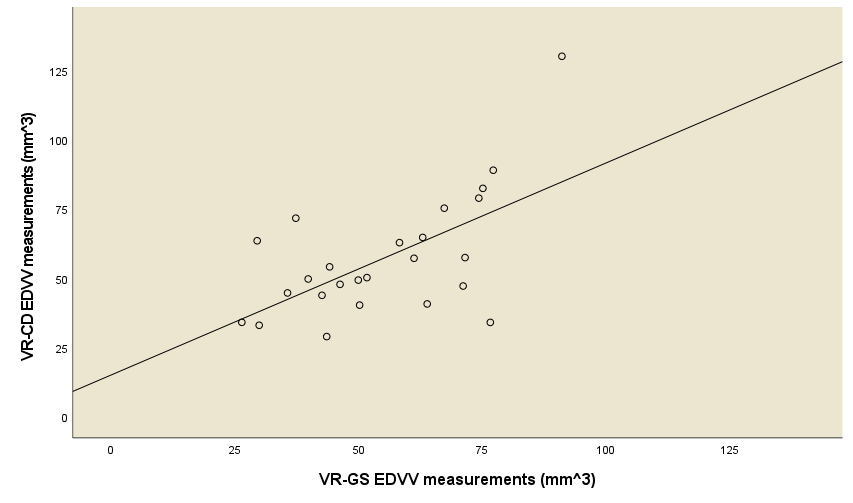

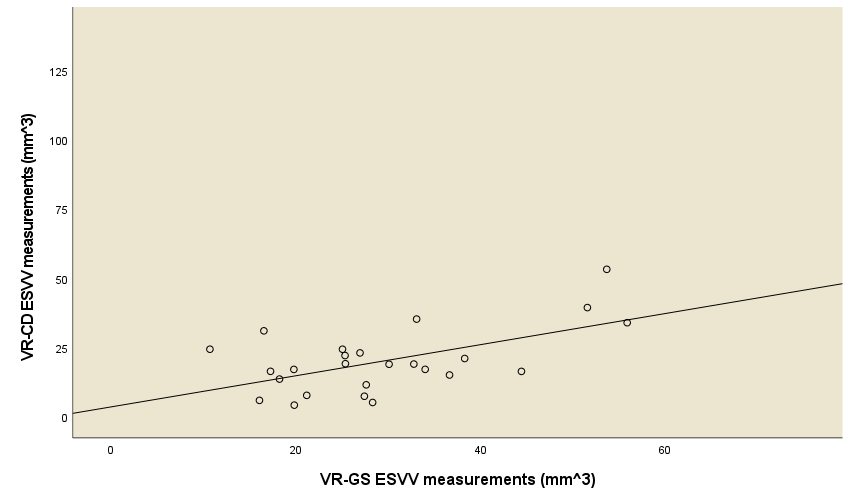


R = 0.58

**VR-GS ESVV measurements (mm^3^)**

**VR-CD ESVV measurements (mm^3^)**

R = 0.61

**VR-GS EDVV measurements (mm^3^)**

**VR-CD EDVV measurements (mm^3^)**

FCVV = fetal cardiac ventricle volume, VR = Virtual reality; VR-CD = VR color Doppler, VR-GS = VR gray-scale, EDVV = end-diastolic ventricle volume; ESVV = end-systolic ventricle volume; VOCAL = Virtual Organ Computer-aided AnaLysis, R = Pearson’s correlation coefficient
