# Supplementary material for: First‐Trimester Fetal Cardiac Function Measurements Using Spatio‐Temporal Image Correlation and Two Ultrasound‐Related Post‐Processing Methods: A Feasibility and Reproducibility Study
Source: Prenat Diagn. 2025 Jul 9;45(9):1130–8. doi: 10.1002/pd.6846 (PMC12322252; doi:10.1002/pd.6846)
Supplement: Supplementary file 2 — Figures S2–S4 [file PD-45-1130-s003.docx]

**Supplemental figure S1.** Protocol first-trimester fetal cardiac ultrasound examination

**2D assessment of the heart**

| Demonstration of cardiac axis (calipers)  Four-chamber view in gray-scale mode  Four-chamber view with CDI*  Left outflow tract with CDI*  Right outflow tract with CDI*  3-vessel view in gray-scale mode  3-vessel view with CDI*  Trachea view in gray-scale mode  Trachea view with CDI*  Tricuspid valve with CDI* and PWD |  |
| --- | --- |

**3D/4D assessment of the heart** Recordings (n) Suggestion angle (degrees)

| STIC in gray-scale mode  STIC with CDI* | 3  3 | 15-20  15-20 |
| --- | --- | --- |

, CDI = color Doppler imaging, , PWD = pulsed-wave Doppler, STIC = Spatio-Temporal Image Correlation, 2D = two-dimensional, 3D = Three-dimensional
*All examinations were performed with strict preset settings for the acquisition of the CDI
