# Supplementary material for: First‐Trimester Fetal Cardiac Function Measurements Using Spatio‐Temporal Image Correlation and Two Ultrasound‐Related Post‐Processing Methods: A Feasibility and Reproducibility Study
Source: Prenat Diagn. 2025 Jul 9;45(9):1130–8. doi: 10.1002/pd.6846 (PMC12322252; doi:10.1002/pd.6846)
Supplement: Supplementary file 3 — Figures S5–S7 [file PD-45-1130-s004.docx]

Supplemental video 1. Demonstration of the clinical application of Virtual Reality (VR) technology through a VR assessment of three-dimensional ultrasound using V-Scope software and the I-Wall.
