# Supplementary material for: First‐Trimester Fetal Cardiac Function Measurements Using Spatio‐Temporal Image Correlation and Two Ultrasound‐Related Post‐Processing Methods: A Feasibility and Reproducibility Study
Source: Prenat Diagn. 2025 Jul 9;45(9):1130–8. doi: 10.1002/pd.6846 (PMC12322252; doi:10.1002/pd.6846)
Supplement: Supplementary file 5 — Figures S5–S7 [file PD-45-1130-s005.docx]

**Supplemental figure S5**. Bland and Altman plots of intra-observer agreement (measurements observer 1 at time point 1 vs. measurements observer 1 at time point 2) with corresponding limits of agreement in proportion of the mean ± 1.96 SD for FCVV measurements (panel A-F).

**A) VR-CD EDVV B) VR-CD ESVV**


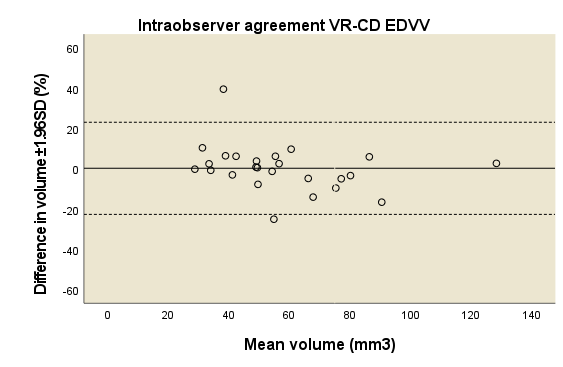

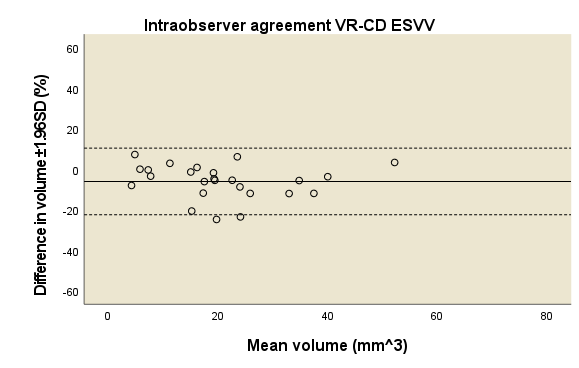


**Difference in volume ±1.96SD (%)**

**Difference in volume ±1.96SD (%)**

**Mean volume (mm^3^)**

**Mean volume (mm^3^)**

**C) VR-GS EDVV D) VR-GS ESVV**


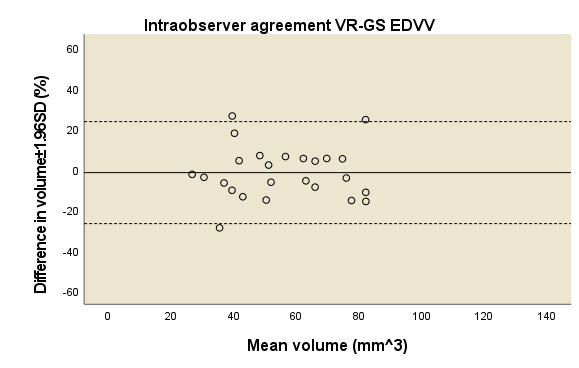

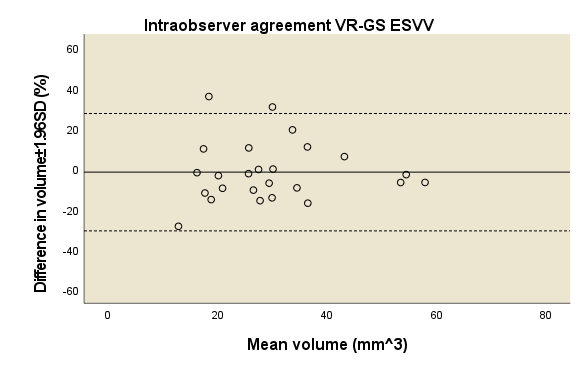


**Mean volume (mm^3^)**

**Mean volume (mm^3^)**

**Difference in volume ±1.96SD (%)**

**Difference in volume ±1.96SD (%)**

**E) VOCAL EDVV F) VOCAL ESVV**


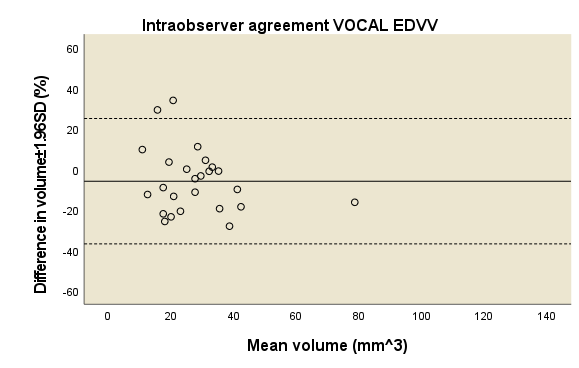

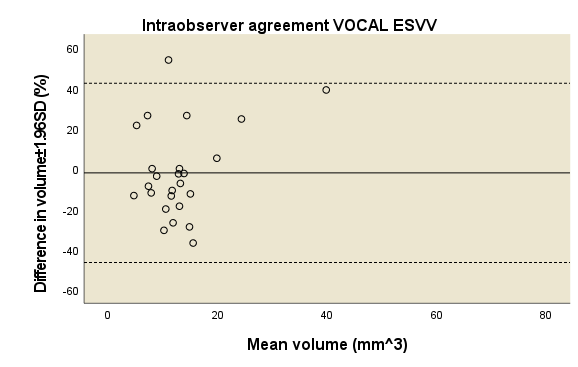


**Difference in volume ±1.96SD (%)**

**Difference in volume ±1.96SD (%)**

**Mean volume (mm^3^)**

**Mean volume (mm^3^)**

FCVV = fetal cardiac ventricle volume, VR = Virtual reality; VR-CD = VR color Doppler, VR-GS = VR gray-scale, EDVV = end-diastolic ventricle volume; ESVV = end-systolic ventricle volume; VOCAL = Virtual Organ Computer-aided AnaLysis

**Supplemental figure S6**. Bland and Altman plots of inter-observer agreement (measurements observer 1 at time point 2 vs. measurements observer 2) with corresponding limits of agreement in proportion of the mean ± 1.96 SD for FCVV measurements (panel A-F).

**A) VR-CD EDVV B) VR-CD ESV**


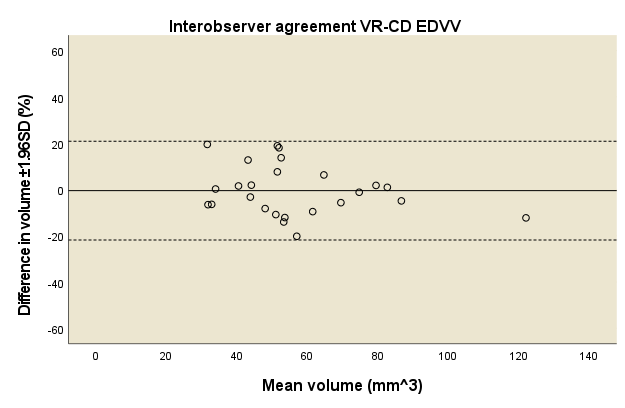

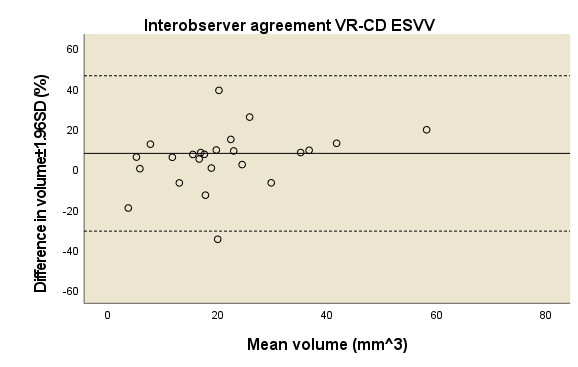


**Difference in volume ±1.96SD (%)**

**Difference in volume ±1.96SD (%)**

**Mean volume (mm^3^)**

**Mean volume (mm^3^)**

**C) VR-GS EDVV D) VR-GS ESVV**


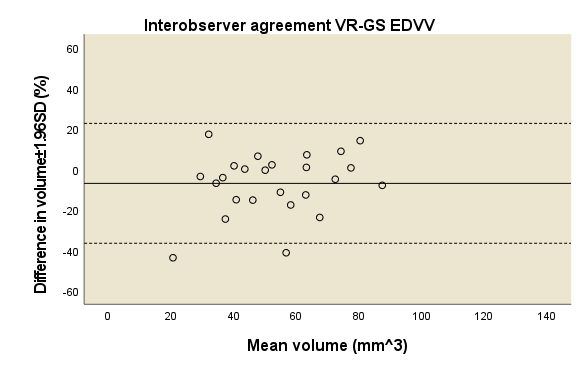

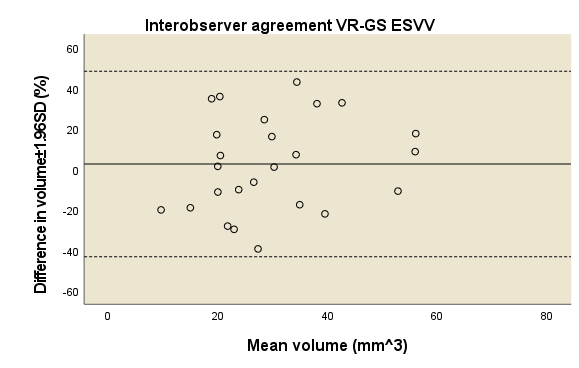


**Difference in volume ±1.96SD (%)**

**Mean volume (mm^3^)**

**Difference in volume ±1.96SD (%)**

**Mean volume (mm^3^)**

**E) VOCAL EDVV F) VOCAL ESVV**


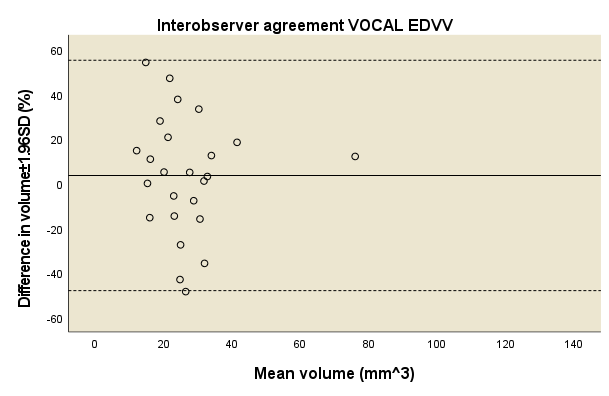

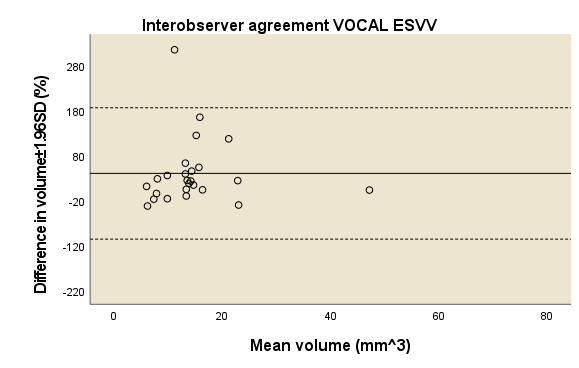


**Mean volume (mm^3^)**

**Mean volume (mm^3^)**

**Difference in volume ±1.96SD (%)**

**Difference in volume ±1.96SD (%)**

FCVV = fetal cardiac ventricle volume, VR = Virtual reality; VR-CD = VR color Doppler, VR-GS = VR gray-scale, EDVV = end-diastolic ventricle volume; ESVV = end-systolic ventricle volume; VOCAL = Virtual Organ Computer-aided AnaLysis

**Supplemental figure S7**. Bland and Altman plots of inter-system agreement (measurements observer 1 at time point 2 vs. measurements observer 1 at time point 2) with corresponding limits of agreement in proportion of the mean ± 1.96 SD for FCVV measurements (panel A-F).

**A) VR-CD EDVV vs. VOCAL EDVV B) VR-CD ESVV vs. VOCAL ESVV**

**
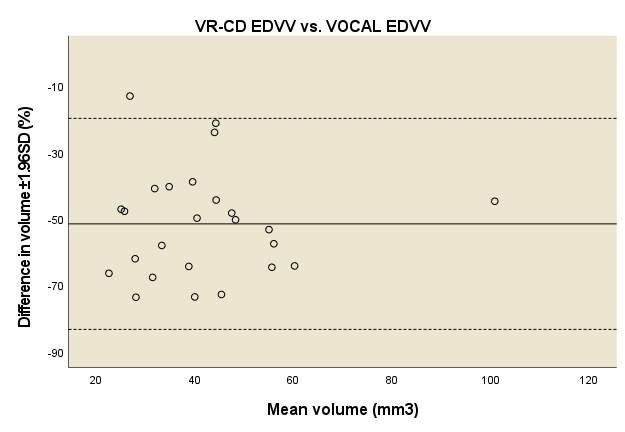

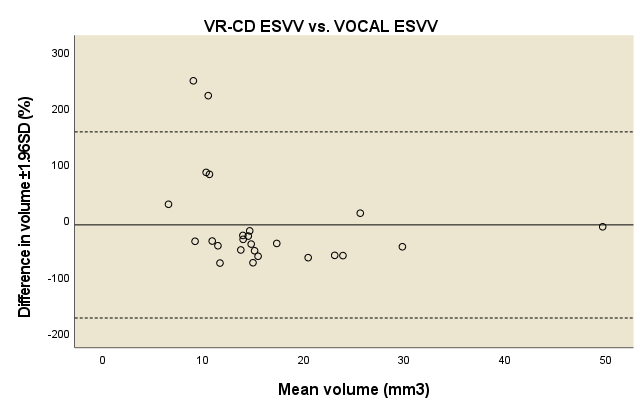
**

**Mean volume (mm^3^)**

**Mean volume (mm^3^)**

**Difference in volume ±1.96SD (%)**

**Difference in volume ±1.96SD (%)**

**C) VR-GS EDVV vs. VOCAL EDVV D) VR-GS ESVV vs. VOCAL ESVV**


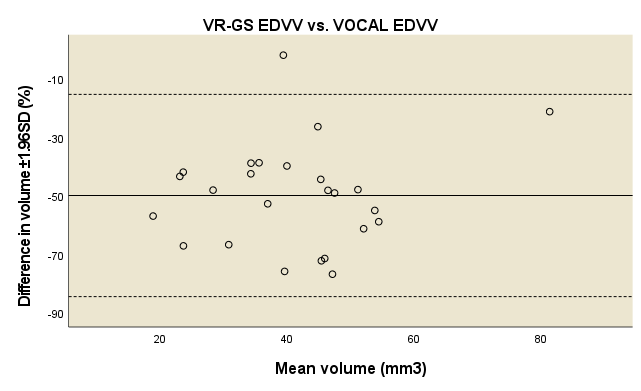

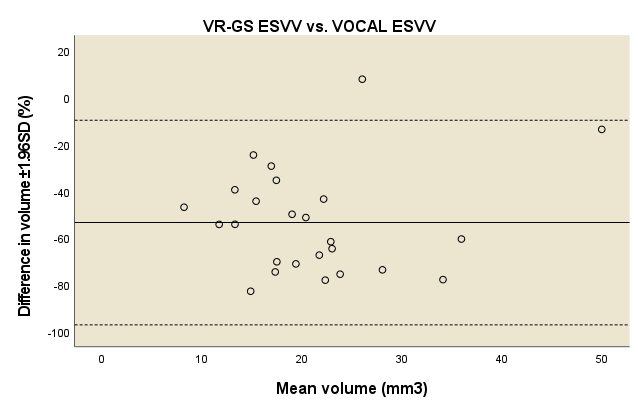


**Mean volume (mm^3^)**

**Mean volume (mm^3^)**

**Difference in volume ±1.96SD (%)**

**Difference in volume ±1.96SD (%)**

**E) VR-CD EDVV vs. VR-GS EDVV F) VR-CD ESVV vs. VR-GS ESVV**

**
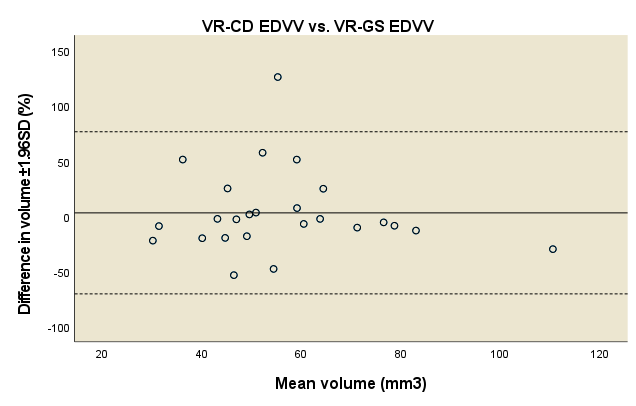

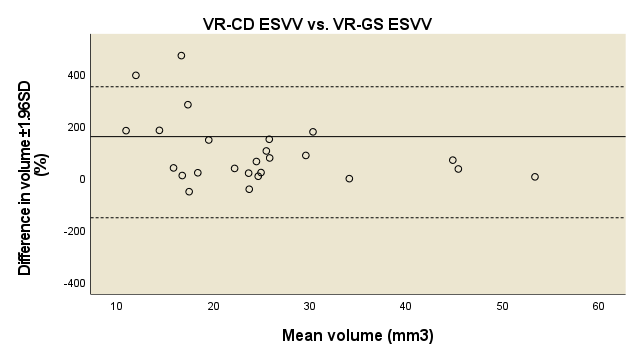
**

**Difference in volume ±1.96SD (%)**

**Mean volume (mm^3^)**

**Difference in volume ±1.96SD (%)**

**Mean volume (mm^3^)**

FCVV = fetal cardiac ventricle volume, VR = Virtual reality; VR-CD = VR color Doppler, VR-GS = VR gray-scale, EDVV = end-diastolic ventricle volume; ESVV = end-systolic ventricle volume; VOCAL = Virtual Organ Computer-aided AnaLysis
